# Antitrypanosomal activity of aloin and its derivatives against *Trypanosoma congolense* field isolate

**DOI:** 10.1186/1746-6148-10-61

**Published:** 2014-03-10

**Authors:** Yitagesu Tewabe, Daniel Bisrat, Getachew Terefe, Kaleab Asres

**Affiliations:** 1 Department of Pharmaceutical Chemistry and Pharmacognosy, School of Pharmacy, Addis Ababa University, Addis Ababa, Ethiopia; 2 Department of Pathology and Parasitology, College of Veterinary Medicine and Agriculture, Addis Ababa University, Debre Zeit, Ethiopia

**Keywords:** African trypanosomiasis, *Aloe gilbertii*, Aloin, Aloe-emodin, Rhein, Antitrypanosomal activities, *Trypanosoma congolense*

## Abstract

**Background:**

There is an urgent need for the development of new, cheap, safe and highly effective drugs against African trypanosomiasis that affects both man and livestock in sub-Saharan Africa including Ethiopia. In the present study the exudate of *Aloe gilbertii*, an endemic *Aloe* species of Ethiopia, aloin, aloe-emodin and rhein were tested for their *in vitro* and *in vivo* antitrypanosomal activities against *Trypanosoma congolense* field isolate. Aloin was prepared from the leaf exudate of *A. gilbertii* by acid catalyzed hydrolysis. Aloe-emodin was obtained by oxidative hydrolysis of aloin, while rhein was subsequently derived from aloe-emodin by oxidation. *In vitro* trypanocidal activity tests were conducted on parasites obtained from infected mice, while mice infected with *T. congolense* were used to evaluate *in vivo* antitrypanosomal activity of the test substances.

**Results:**

Results of the study showed that all the test substances arrested parasites motility at effective concentration of 4.0 mg/ml within an incubation period ranging from 15 to 40 min. Moreover, the same concentration of the test substances caused loss of infectivity of the parasites to mice during 30 days observation period. Among the tested substances, rhein showed superior activity with minimum inhibitory concentration (MIC) of 0.4 mg/ml. No adverse reactions were observed when the test substances were administered at a dose of 2000 mg/kg. Rhein at doses of 200 and 400 mg/kg, and the exudate, aloin and aloe-emodin at a dose of 400 mg/kg reduced the level of parasitaemia significantly (*P* < 0.05) and improved anaemia.

**Conclusion:**

The results obtained in this investigation indicate that aloin and its derivatives particularly rhein have the potential to be used as a scaffold for the development of safe and cost effective antitrypanosomal drugs that can be useful in the continuing fight against African trypanosomiasis.

## Background

Trypanosomiasis is a potentially fatal human and animal disease caused by the parasitic protozoa of the genus *Trypanosoma*. These diseases occur in tropical Africa and South America [1,2]. Human African trypanosomiasis (HAT) or sleeping sickness is a severe fly-borne disease caused by protozoa of the species *Trypanosoma brucei rhodesiense* and *T.b. gambiense*. This disease was first described by the late 1800s and early 1900s even if it had probably existed in Africa many centuries before. The disease occurs in vast geographical regions ranging from the Sahara to the Kalahari Desert [1,3]. Until recently, African trypanosomiasis was receiving very few attentions, and health interventions and research and development were inadequate to the need [4-6].

African animal trypanosomosis (AAT) is also a major constraint to livestock productivity. It has a significant impact on the livelihood of millions of people in Africa and costing several billion US dollars each year [5]. *T. congolense*, *T. vivax* and, to a lesser extent *T. b. brucei*, are the agents of trypanosomosis in livestock [1,7]. In Ethiopia, it has been described as a major impediment to the livestock development and agricultural production [8].

The current options for trypanosomiasis treatment are ineffective due to several reasons, making the search for improved drugs essential. Moreover, because those in the lower socioeconomic class are disproportionately affected by the disease, there is a great need for the development of not only less toxic and more effective drugs but also less expensive agents [6,9].

Aloin and its derivatives are found in large amounts in the leaf exudates of several *Aloe* species of Ethiopia such as *Aloe gilbertii* T.Reynolds. The investigation of the biological properties of aloin and its semi-synthetic derivatives could prove to be invaluable from a medicinal perspective. Hence, this study was conducted to evaluate the possible antitrypanosomal activity of these plant products which were obtained from the robust and easily cultivatable endemic *Aloe* species of Ethiopia.

## Materials and methods

### Materials

#### Plant material

Leaves of *A. gilbertii* were collected from Oromia region, Shashemene Woreda, Jello Kebele, some 250 km south of Addis Ababa, Ethiopia. Identification and authentication of the plant were made by Prof. Sebsebe Demissew at the National Herbarium, Department of Biology, Addis Ababa University where a voucher specimen was deposited (collection number AG 001).

#### Instruments and apparatus

Homemade silica gel thin layer chromatography (TLC) plates (Merck, Germany) of 0.25 mm thickness were used for isolation and analytical chromatographic purposes. ^1^H NMR, ^13^C NMR spectra were recorded on Bruker Avance DMX400 FT-NMR spectrometer operating at 400 MHz and 100 MHz, respectively, at room temperature using deuterated methanol or chloroform. ESI-MS were measured on Ultimate 3000 LC-MS using an electrospray ionization method with negative mode. The source voltage and temperature were fixed at 3 kV and 250°C.

#### Test organisms

*T. congolense* stocks that were originally isolated from a pure natural infection of cattle herd in Arbaminch area, southwest Ethiopia were obtained from School of Veterinary Medicine, Addis Ababa University. The organisms were maintained by serial passages in mice until required.

#### Experimental animals

Swiss albino mice of either sex, weighing 25-35 g (age 8-12 weeks) were obtained from the College of Veterinary Medicine and Agricultture, Addis Ababa University. The animals were acclimatized for a period of 7 days at room temperature 23-25°C with relative humidity of 60-65% and allowed free access to pellet diet and water *ad libitum*. All procedures complied with the Guide for the Care and Use of Laboratory Animals [10] and approved by the Institutional Review Board of the School of Pharmacy, Addis Ababa University.

### Methods

#### Preparation of test substances

##### Exudate

Exudate was collected from the leaves of *A. gilbertii* by arranging the leaves concentrically around a depression in the soil, which was covered with a plastic sheet. It was then left in open air for 3 days to allow evaporation of water, which yielded a yellow powder.

##### Aloin

The exudate (10 g) was dissolved in methanol, filtered and dried in a rotary evaporator under reduced pressure. The resulting powder was then dissolved in methanolic HCl (2%) and heated to 60°C under reflux for 2 h until a single spot was obtained when monitored by TLC. After removing of the solvent under reduced pressure, the reaction product was subjected to preparative thin layer chromatography (PTLC) over silica gel and developed using chloroform: methanol (4:1) as a solvent system to obtain a yellow amorphous solid [*R*_*f*_ = 0.35 (CHCl_3_/ MeOH; 4:1), (-)LR-ESIMS *m/z* = 417 [M-H]^−^, C_21_H_21_O_9_; ^1^H NMR (400 MHz, CDCl_3_) δ 11.88/11.85 (1-OH, *brs*), 11.80/11.78 (8-OH, *brs*), 7.50/7.57 (H-6, *t*), 7.06/7.08 (H-5, *d*), 7.01/7.03 (H-4, *d*), 6.88/6.90 (H-7, *d*), 6.83/6.85 (H-2, *d*), 4.95/4.96 (H-15, *s*), 4.56/4.57 (H-10, *s*), 2.72-3.99 (H-1'-H-6'; Glucose). ^13^C NMR (100 MHz, CDCl_3_) δ 193.82/193.77 (C-9), 161.56 (C-8), 161.33/161.21 (C-1), 152.65/151.82 (C-3), 146.30/146.09 (C-12), 142.47/142.26 (C-14), 136.54/135.67 (C-6), 120.70/119.36 (C-5), 118.27/116.67 (C-4), 117.82/117.50 (C-11), 116.26/116.17 (C-13), 116.07/115.84 (C-7), 113.10/112.78 (C-2), 62.86/62.82 (C-15), 44.62/44.32 (C-10), 60.21-85.61 (C-1'-C-6' glucose)] identified as aloin [11].

##### Aloe-emodin

Aloin (about 1 g) was added to an acidic solution containing a mixture of 25 ml of concentrated hydrochloric acid and 75 ml of water. 50 ml of a 20% aqueous solution of ferric chloride were added to the above solution and the resulting mixture was transferred to a round bottom flask. About 30 ml of toluene were added to the above mixture and the biphasic mixture refluxed for 8 h at 100°C. The reaction mixture was then allowed to cool to about 90°C and the organic layer was separated and kept overnight at 8°C to yield aloe-emodin [Orange solid crystals, (-)HR-ESIMS *m/z =* 269.0452 [M-H]^−^, C_15_H_9_O_5_, (calc. = 269.0450), ^1^H NMR (400 MHz, DMSO-*d*_6_): δ 11.80 (1-OH and 8-OH, *brs*), 7.70 (H-6, *t*), 7.65 (H-5, *dd*), 7.60 (H-4, *brs*), 7.30 (H-7, *dd*), 7.20 (H-2, *brs*), 5.60 (11-OH, *brs*), 4.60 (H-11, *s*). ^13^C NMR (100 MHz, DMSO-*d*_6_): 191.37 (C-9), 181.02 (C-10), 161.54 (C-8), 161.23 (C-1), 153.62 (C-3), 137.18 (C-6), 133.01 (C-12), 132.77 (C-14), 124.22 (C-7), 120.51 (C-2), 119.19 (C-5), 116.97 (C-4), 115.53 (C-11), 114.08 (C-13), 61.99 (C-15) [12-14].

##### Rhein

**Scheme 1:**
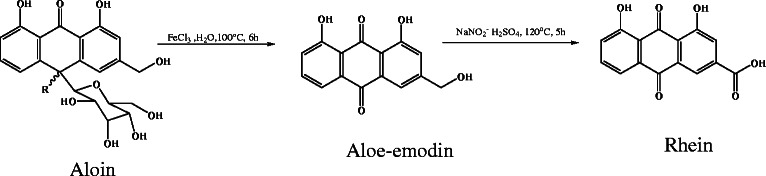
Oxidative hydrolysis of aloin to aloe-emodin and further oxidation of aloe-emodin to rhein.

Sodium nitrite (1.275 g) dissolved in 7 ml of conc. sulfuric acid was heated to about 120°C. Aloe-emodin (0.5 g) was added in small amount to this mixture over a period of 30 min. The reaction was allowed to continue for 5 h and the reaction mixture poured into ice to give an orange brown precipitate. The precipitate formed was filtered and then dissolved in sodium carbonate solution (pH not exceeding 9.5) and extracted with toluene to remove the unreacted aloe-emodin. The aqueous solution was treated with concentrated hydrochloric acid and the precipitate filtered, washed, dried and recrystallized from methanol to obtain a pale yellow powder identified as rhein [(-)HR-ESIMS *m/z* = 283.0248 [M-H]^−^, (calc. 283.0243), ^1^H NMR (400 MHz, DMSO-*d*_6_): δ 11.95 (1-OH and 8-OH, *brs*), 10.20 (11-COOH, *brs*), 8.20 (H-4), 7.75 (H-2), 7.65 (H-6), 7.60 (H-5), 7.40 (H-7)] (Scheme 1) [13,14].

##### Acute toxicity study

Acute toxicity study was carried out using to OECD guideline for testing of chemicals using Swiss albino mice [15]. Twenty-four female Swiss albino mice were randomly divided into 4 groups of 6 mice per cage. Before administration of a single dose of the test substance, the mice were fasted for 2 h (water allowed) [16]. Each group of mice separately received 2000 mg/kg of the test substance orally and were observed closely during the first 30 min after dosing, periodically during the first 24 h with special attention to the first 4 h and once daily thereafter for a total of 7 days. The experimental animals were monitored for signs of toxicity such as changes in skin colour, blinking eyes, tremors, convulsion, lacrimation, muscle weakness, sedation, urination, salivation, diarrhoea, lethargy, sleep, coma and death, if any.

### *In vitro* antitrypanosomal activity test

Assessment of *in vitro* trypanocidal activity was performed in triplicate in 96 well microtiter plates. Infected mice with high parasitaemic state were sacrificed and the blood collected in EDTA coated syringe prepared with phosphate buffer saline glucose (PBSG). Blood (20 μl) containing about 20-25 parasites per field was mixed with 5 μl of each of the test substances dissolved in 10% DMSO in concentrations of 20.0, 10.0, 2.0 and 0.5 mg/ml to produce effective test concentrations of 4.0, 2.0, 0.4 and 0.1 mg/ml, respectively [17-20]. To a set of controls containing the parasite (20 μl of infected blood) suspended in 10% DMSO only, and a similar concentration of standard trypanocidal drug diminazene aceturate were included to serve as negative and positive controls, respectively [17,19-21].

After 5 min incubation in closed microtiter plates maintained at 37°C, about 20 μl of test mixtures were placed on separate microscope slides and covered with 7×22-mm cover slips and the parasites were observed every 5 min for death/motility for a total duration of one h using X400 objective.

### *In vivo* infectivity test

Infectivity test was performed in order to know if there are any remaining parasites after *in vitro* test which are infective. In this test the inoculum contained each remaining incubation mixture from each well of the micro titer plate where the *in vitro* test was carried out i.e. 4, 2, 0.4 and 0.1 mg/ml of test substance separately in 0.02 ml of infected blood as described by Yusuf *et al.*[6] and Maikai [19]. A total of 105 healthy mice (5 animals per dose) received each mixture intraperitoneally (after 2 h incubation) and observed for 30 days for the development of infection.

### *In vivo* antitrypanosomal activity test

#### Parasite inoculation and test compounds administration

A total of ninety healthy mice were randomly grouped into fifteen groups (A-I, A-II, A-III, B-I, B-II, B-III, C-I, C-II, C-III, D-I, D-II, D-III, and E, F, G) of six mice each. All experimental groups except group G were then infected intraperitoneally with 0.2 ml of infected blood diluted with PBS containing approximately 10^5^ trypanosome cells collected from donor mice by cardiac puncture.

The animals were allowed to develop parasitaemia in 14 days, and treatment was initiated on day 15 when parasites were detected and average parasitaemia became approximately 7.20 (log number) ~10^7.20^/ml. Groups A-I, A-II, A-III, B-I, B-II, B-III, C-I, C-II, C-III and D-I, D-II, D-III were injected intraperitoneally 100, 200 and 400 mg/kg of the leaf exudate, aloin, aloe-emodin and rhein, respectively. Dose selection for each group was made after undertaking oral acute toxicity study. The test substances dissolved in 10% DMSO were administered to the experimental mice every morning for seven days. Group E (positive control group) received 28 mg/kg (intraperitoneal single dose) of the standard drug diminazene aceturate dissolved and reconstituted in distilled water. Group F (negative control group) were infected with the parasite but not treated, whereas Group G included uninfected-untreated mice for reference purpose e.g. for comparison of some haematological parameters.

#### Determination of parastaemia

Parasitaemia was monitored on every other day by microscopic examination of blood obtained from the tail and pre-sterilized with methylated spirit. The degree of parastaemia was determined microscopically at X400 magnification using the “Rapid Matching” method as outlined by Herbert and Lumsden [22]. The method involves microscopic counting of parasites per 1, 5, 10 and 20 fields in pure blood or blood appropriately diluted with PBS (pH 7.2). Wet smear was prepared in triplicate from each animal and the mean value of slide counts was taken per sample examined microscopically. Logarithm values of these counts were obtained by matching with the Table of Herbert and Lumsden [22].

Daily treatment continued for seven days with continuous monitoring of parasitaemia every other day until the 14^th^ day. For the assessment of antitrypanosomal effect of the test compounds, the level of parasitaemia (expressed as log of absolute number of parasites per milliliter of blood) in the treated animals was compared with those of the control animals [19,23].

#### Determination of packed cell volume (PCV)

Packed cell volume (PCV) was monitored on days 0, 7 and 14 using Wintrobe’s method. Heparinised capillary tubes were filled two-thirds to three-quarters with blood obtained by bleeding tail vein of mice and sealed immediately. The tubes were then centrifuged in a micro-centrifuge for 5 min at 10000 rpm. After centrifugation, the height of the red blood cell column was measured by the use of haematocrit reader and compared to the total height of the column of the whole blood.

#### Statistical analysis

The data were analyzed using Statistical Package for Social Science (SPSS), version 16.0. Values of the data obtained from the study were summarized and expressed as mean ± standard error of mean (SEM). One way ANOVA followed by Tukey’s multiple comparison tests was performed to determine statistical significance. The level of significance for the differences between means within a group and *in vitro* data were computed by student’s *t* test. P values less than 0.05 were considered significant.

## Results and discussion

The results of acute toxicity study revealed that none of the tested substances possesses adverse reactions like increased motor activity, blinking eyes, tremors, convulsion, lacrimation, stimulation, muscle weakness, sedation, urination, salivation, lethargy, sleep, tremors, arching and rolling and coma up to a dose of 2,000 mg/kg. However, minor signs of toxicity such as temporary hair erection and diarrhoea were observed in two of the experimental animals. This indicates that the test substances are safe at the dose levels used and the LD_50_ of the test substances are above 2 g/kg as per the OECD guideline [15]. Initially, preliminary antitrypanosomal activity screening was conducted by an *in vitro* method, which is relatively simple, cheap and reliable. As reported previously [17,19,24], under this *in vitro* system, trypanosomes could survive for up to 4 h or more in the absence of drugs. Parasites motility constitutes a relatively reliable indicator of viability among most zooflagellate parasites and cessation or drop in motility of the parasites in test substances treated blood compared to that of parasite-loaded control blood without test substances was taken as a measure of trypanocidal activity [17,20]. The *in vitro* test showed that at a concentration of 4.0 mg/ml the parasites were completely immobilized or killed by incubation with the leaf exudate*,* aloin, aloe-emodin and rhein within 25, 40, 20 and 15 min, respectively, while the standard drug diminazene aceturate immobilized or eliminated the parasites within 10 min of incubation at the same concentration (Table 1). The control consisting of trypanosomes incubated with 10% DMSO showed the presence of very active parasites for more than 1 h. In agreement with previous reports [21,25,26], the present results confirm that compounds with anthraquinone scaffold exhibit *in vitro* antitrypanosomal activity. Previously, aloe-emodin has been reported to have activity (IC_50_ = 14 μM) against the bloodstream form of *T.b. brucei*[27]. According to Atawodi [28], the mean minimum inhibitory concentration (MIC) value of the common trypanocidal drugs is 10.7 mg/ml, and that agents with MIC values of 5 - 20 mg/ml could be regarded as very active. Therefore, the exudate and pure compounds tested in the present study can be considered as promising antitrypanosomal agents, although their activity was not as strong as that of the positive control diminazene aceturate. However, the activity of the compounds may be increased by structural modification.

**Table 1:** **Effect of test substances on motility of *Trypanosoma congolense***

**Test substance**	**Time (min) after which motility ceased vs. effective concentrations (mg/ml)**				**Parasite motility**
	**4.0**	**2.0**	**0.4**	**0.1**	
Exudate	25	40	55	>60	++
Aloin	40	60	>60	>60	+
Aloe-emodin	20	40	55	>60	++
Rhein	15	25	45	>60	+++
Diminazene aceturate	10	20	40	>60	+++
10% DMSO	NE	NE	NE	NE	NE

+++ = reduced highly, ++ = reduced moderately, + = reduced slightly, DMSO = dimethyl sulfoxide, NE = no noticeable effect on motility even after 60 min.

It should be noted that in the *in vitro* model, complete immobility of parasites does not necessarily mean that the parasites are dead, but rather the parasites may have lost their infectivity. This was confirmed through the infectivity test which showed that the test substances inhibited healthy mice from developing infection for more than 30 days or significantly prolonged the prepatent period especially at a concentration of 4.0 mg/ml (Table 2) unlike the negative control which developed infection within 11 days after inoculation. The test substances may have caused loss of infectivity by abrogating some vital metabolic processes in the parasites or inducing some morphological changes in the parasites that render them more susceptible to the mice immune defense systems. In the present *in vivo* study, there was a reduction in parasitaemia following administration of the leaf exudate as well as the pure compounds. The results confirmed that all the test substances possess mild to moderate antitrypanosomal activity *in vivo*, but did not completely clear the parasite. The inability of the test substances to clear the parasite from the blood could be because of their failure to reach the site of action or rapid metabolization [29]. Though parasitaemia was not completely eliminated, rhein was found to be the most active of all the substances tested (Table 3). Also, at a dose of 400 mg/kg the leaf exudate of *A. gilbertii*, reduced parasitaemia level significantly.

**Table 2:** **Duration (days) after which parasitaemia developed in mice inoculated with a mixture of test substances and *Trypanosoma congolense *** **infected blood**

**Dose of test substance mixed with 0.02 ml of infected blood**	**Number of mice which developed infection**	**Infection interval in days (mean ± SEM)**
4.0 mg/ml E	0/5	Ni
2.0 mg/ml E	2/5	17.50 ± 0.50
0.4 mg/ml E	4/5	13.75 ± 0.48
0.1 mg/ml E	5/5	12.20 ± 0.40
4.0 mg/ml A	1/5	26.00
2.0 mg/ml A	3/5	16 ± 0.58
0.4 mg/ml A	5/5	13.40 ± 0.24
0.1 mg/ml A	5/5	11.80 ± 0.66
4.0 mg/ml AE	0/5	Ni
2.0 mg/ml AE	2/5	18.00 ± 1.00
0.4 mg/ml AE	4/5	14.00 ± 0.40
0.1 mg/ml AE	5/5	12.00 ± 0.54
4.0 mg/ml Rx	0/5	Ni
2.0 mg/ml Rx	2/5	19.50 ± 0.71
0.4 mg/ml Rx	3/5	17.00 ± 0.58
0.1 mg/ml Rx	5/5	14.20 ± 0.37
4.0 mg/ml DA	0/5	Ni
2.0 mg/ml DA	1/5	19.00
0.4 mg/ml DA	3/5	16.33 ± 0.33
0.1 mg/ml DA	5/5	13.40 ± 0.81
0.1 ml 10% DMSO	5/5	11.20 ± 0.49

N = 5, Ni = no infection developed in the observation period, E = leaf exudate of *Aloe gilbertii*, A = aloin, AE = aloe-emodin, Rx = rhein, DA = diminazene aceturate, DMSO = dimethylsulfoxide, SEM = standard error of mean.

**Table 3:** **Effect of test substances on parasitaemia level of *Trypanosoma congolense *** **infected mice**

**Test substance**	**Dose (mg/kg)**	**Trypanosomes/ml**^**a**^							
		** D0**	** D2**	** D4**	** D6**	** D8**	** D10**	** D12**	** D14**
Exudate	100	7.76 ± 0.02	7.84 ± 0.06	8.00 ± 0.09	7.96 ± 0.08	7.18 ± 0.38	7.64 ± 0.12	7.76 ± 0.18	8.18 ± 0.09
	200	7.50 ± 0.10	7.90 ± 0.08	7.80 ± 0.06	7.14 ± 0.24	6.06 ± 0.11	7.06 ± 0.29	7.56 ± 0.15	7.82 ± 0.15
	400	7.46 ± 0.09	7.86 ± 0.10	7.90 ± 0.15	4.92 ± 1.23^b^	3.84 ± 0.90^b^	4.56 ± 1.20^b^	4.80 ± 0.15^b^	5.44 ± 0.37
Aloin	100	7.34 ± 0.06	7.70 ± 0.13	7.96 ± 0.08	8.07 ± 0.07	7.54 ± 0.21	7.70 ± 0.14	7.80 ± 0.18	8.19 ± 0.08
	200	7.74 ± 0.05	7.94 ± 0.06	7.86 ± 0.05	7.40 ± 0.29	6.60 ± 0.32	7.10 ± 0.27	7.82 ± 0.18	7.83 ± 0.13
	400	7.38 ± 0.09	7.95 ± 0.14	7.97 ± 0.11	5.24 ± 1.40	4.38 ± 1.09^b^	5.62 ± 1.50	5.98 ± 0.15	6.02 ± 0.49
Aloe-emodin	100	7.48 ± 0.09	7.72 ± 0.73	7.96 ± 0.05	8.14 ± 0.17	7.57 ± 0.19	7.75 ± 0.08	7.82 ± 0.17	8.20 ± 0.05
	200	7.42 ± 0.07	7.92 ± 0.03	7.88 ± 0.03	7.48 ± 0.30	6.77 ± 0.27	7.36 ± 0.20	7.76 ± 0.16	7.97 ± 0.17
	400	7.37 ± 0.08	7.90 ± 0.15	8.00 ± 0.13	5.38 ± 1.42	4.56 ± 1.14^b^	6.04 ± 1.52	6.74 ± 0.13	6.90 ± 0.38
Rhein	100	7.23 ± 0.18	7.29 ± 0.27	6.49 ± 0.30	5.58 ± 0.40	4.92 ± 1.53	4.22 ± 1.56	5.21 ± 0.37	5.57 ± 1.51
	200	7.41 ± 0.08	6.97 ± 0.33	5.32 ± 1.37	5.24 ± 1.35	3.66 ± 1.51^b^	4.10 ± 0.27^b^	4.82 ± 0.18^b^	4.83 ± 0.13^b^
	400	7.41 ± 0.08	7.00 ± 0.25	4.88 ± 1.28^b^	3.66 ± 1.50^b^	2.34 ± 1.44^b^	2.22 ± 1.45^b^	2.96 ± 0.33^b^	3.22 ± 0.25^b^
Deminazene aceturate	28	7.35 ± 0.08	0.00 ± 0.00	0.00 ± 0.00	0.00 ± 0.00	0.00 ± 0.00	0.00 ± 0.00	1.08 ± 1.08	1.97 ± 1.36
Distilled water	0.1 ml	7.43 ± 0.10	8.10 ± 0.08	8.60 ± 0.08	8.65 ± 0.13	8.71 ± 0.06	9.36 ± 0.20	8.94 ± 0.03	8.72 ± 0.34

^a^The antilog values of the numbers indicated in the table give the absolute number of trypanosomes/ml of blood; ^b^Significantly different when compared with the negative control (*P* < 0.05); Values are Mean ± SEM; N = 6; D = day; D0 = the day treatment commenced; SEM = standard error of mean.

From the results of the present study it is not possible to know the mechanism (s) by which the test substances exert their antitrypanosomal activity. However, accumulated evidence [25,28] suggests that many natural products which contain anthraquinone scaffold exhibit their antitrypanosomal activity by virtue of their interference with the redox balance of the parasites acting either on the respiratory chain or on the cellular defenses against oxidative stress. This is because anthraquinones are capable of generating radicals that may cause peroxidative damage to trypanothione reductase that is very sensitive to alterations in redox balance.

The experiment on PCV analysis also gave results that were consistent with the observations made on parasitaemia levels. Anaemia is the most outstanding clinical and laboratory feature of African trypanosomiasis and also the primary cause of death. Anaemia as indicated by PCV level is known to worsen with increasing parasitaemia [30]. As shown in Table 4, the PCV at days 7 and 14 of mice treated with 400, 200 and 100 mg/kg of rhein was on average above 45%, which was within the range of reference values (42-52%). Similarly the PCV of animals treated with 400 mg/kg of the other test substances was found to be above 46% after 14 days of treatment which is significantly different (*P* < 0.05) when compared to the negative control. This improvement of PCV may be due to reduction of the parasitaemia level or neutralizing the toxic metabolites produced by trypanosomes after treatment.

**Table 4:** **Effect of test substances on packed cell volume (PCV) of *Trypanosma congolense *** **infected mice**

**Treatment**	**Dose (mg/kg)**	**PCV/Day of treatment**		
		** PCV0**	** PCV7**	** PCV14**
Exudate	100	47.10 ± 0.68	42.40 ± 1.07^bc^	45.10 ± 0.91^a^
	200	48.30 ± 1.11	44.00 ± 1.50^b^	48.20 ± 0,46^a^
	400	47.00 ± 1.21	43.60 ± 1.41^b^	47.60 ± 1.44^a^
Aloin	100	46.40 ± 1.53	40.00 ± 1.87^bc^	42.70 ± 1.21^bc^
	200	46.00 ± 1.17	40.60 ± 1.22^bc^	43.90 ± 0.66^bc^
	400	45.50 ± 1.50	41.70 ± 1.21^bc^	46.20 ± 1.34^a^
Aloe-emodin	100	50.20 ± 1.20	44.20 ± 1.65^b^	47.20 ± 0.73^a^
	200	47.90 ± 0.60	44.30 ± 0.80^b^	46.10 ± 0.87^a^
	400	47.10 ± 0.57	45.90 ± 1.45^b^	47.30 ± 0.73^a^
Rhein	100	47.10 ± 0.64	45.40 ± 0.76^b^	47.20 ± 0.25^a^
	200	50.00 ± 0.65	45.80 ± 0.46^b^	49.30 ± 0.41^a^
	400	50.20 ± 1,10	48.00 ± 1.15	50.40 ± 0.73^a^
Diminazene aceturate	28	50.10 ± 0.33	48.60 ± 0.57	50.60 ± 0.43^a^
Distilled water	0.1 ml	46.00 ± 0.79	42.10 ± 1.18^bc^	40.10 ± 1.10^*^
Uninfected untreated	_	50.50 ± 0.59	51.10 ± 0.66	50.80 ± 0.67

Values are Mean ± SEM, N = 6, D = day, PCV0 = PCV on the day treatment commenced, SEM = standard error of mean. All superscripts indicate significance at *P* < 0.05 (^a^against negative control, ^b^against uninfected-untreated, ^c^against 28 mg/kg diminazene aceturate, ^*^against all groups), PCV was compared with each other mainly at days 7 and 14.

## Conclusion

The results of the present study revealed that the leaf exudate of *A. gilbertii* as well as the pure compounds tested, namely aloin, aloe-emodin, and rhein possess mild to moderate antitrypanosomal activity both *in vitro* and *in vivo* against *T. congolense* field isolate. More importantly, the work confirmed that the two anthraquinones, namely aloe-emodin and rhein, particularly the latter, possess a much better activity than aloin which contains the anthrone moiety. Thus, the promising activity profile of the anthraquinones tested along with their relative margin of safety merit the use of these compounds as leads for the development of safer, more potent and cost effective alternative drugs for the treatment of African trypanosomiasis.

## Competing interests

The authors declare that they have no competing interests.

## Authors’ contributions

YT: Collected plant material, carried out the experimental work and drafted the manuscript. DB: Assisted in the extraction, isolation, preparation and identification of compounds including interpretation of spectral data. GT: Designed experiments for both *in vitro* and *in vivo* antitrypanosomal activity testing. KA: Involved in the isolation and preparation of compounds, corrected, edited and proof read the manuscript before submission. All authors read and approved the final manuscript.
